# The strategic concept for the Lithuanian bioeconomy: insights for niche bioenergy sectors

**DOI:** 10.12688/openreseurope.16085.1

**Published:** 2023-06-20

**Authors:** Vlada Vitunskienė, Akvilė Aleksandravičienė, Jonas Čaplikas, Agnė Dapkuvienė

**Affiliations:** 1 Vytauto Didziojo Universitetas, Kaunas, Kaunas County, LT-44248, Lithuania

**Keywords:** Bioeconomy, biofuels, biogas, energy dependency, Lithuania

## Abstract

This article describes a strategic concept for the Lithuanian bioeconomy that was developed as a deliverable of the Task 1.5 in the BIOEAST’sUP H2020 project. We aimed to create a conceptual basis for the preparation of a national bioeconomy strategy and/or action plan in order to initiate a deeper discussion about the strategically oriented development of a knowledge-based circular bioeconomy in Lithuania. Our strategic insights are focused on two niche energy sectors like biofuels and biogas. The results of the SWOT analysis reveal that, in Lithuania, a synthesis of the biofuels and biogas sectors' strategic directions is necessary to develop over-arching national bioeconomy-appropriate strategic actions, such as market intervention actions, research, innovation and education actions, as well as governance and policy actions.

## Disclaimer

The views expressed in this article are those of the author(s). Publication in Open Research Europe does not imply endorsement of the European Commission.

## Introduction

Global challenges such as climate change, land, and ecosystem degradation, coupled with a growing population, force us to seek for new ways of production and consumption that respect the ecological boundaries of our planet. To overcome these challenges, we need to improve and update the way we produce and consume food, products, and materials within healthy ecosystems through a sustainable bioeconomy. The bioeconomy can be a catalyst for sustainable systemic change and transition, tackling key economic, societal and environmental challenges faced by EU countries ^1^.

The European Commission defines the bioeconomy as an economy that “encompasses the production of renewable biological resources and the conversion of these resources and waste streams into value added products, such as food, feed, bio-based products and bioenergy” [ 2, p. 3]. This is the official definition used in Lithuania.

The main goal of this article is to present a conceptual basis for the preparation of a national bioeconomy strategy and/or action plan to initiate a deeper discussion about the strategically oriented development of a knowledge-based circular bioeconomy in Lithuania. An analysis of policies and institutions related to the state of the bioeconomy was performed. The concept foresees the need for and importance of intervention and strategic directions in bioeconomy sectors for the country, such as the production of biogas and biofuels. This article was prepared based on the statistical analysis of various information sources, studies and documents, as well as the SWOT analysis of the bioeconomy through experts’ assessments to support strategic planning for promising niche bioenergy sectors.

## Bioeconomy-related institutional framework in Lithuania

**Figure 1.**
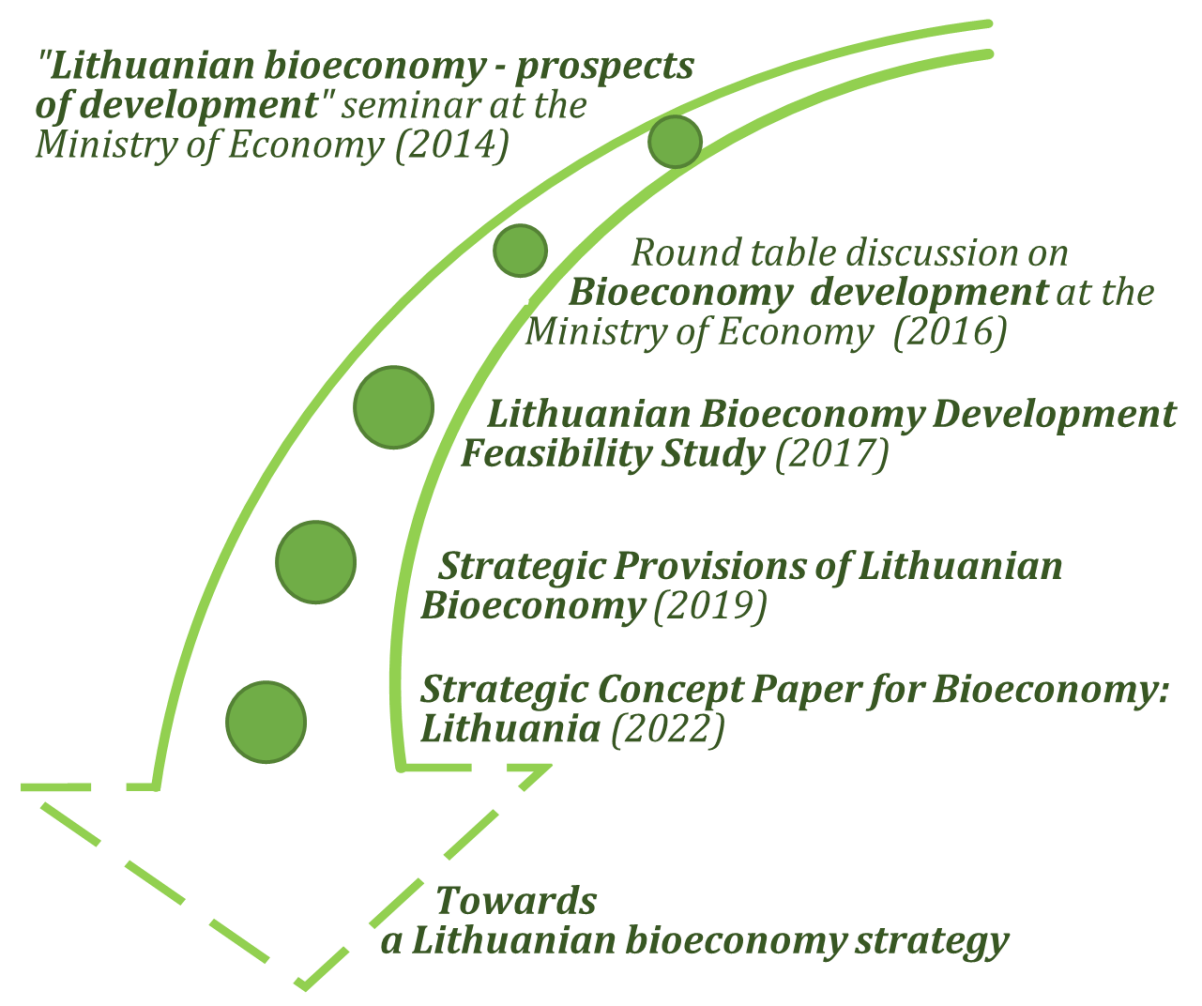
Lithuania’s path towards the bioeconomy strategy.

Lithuania does not have a national bioeconomy strategy and none of them has an action plan, despite its high biomass resource base and modern biorefining potential in many cases, as revealed in both studies Lithuanian Bioeconomy Development Feasibility Study ^3^ and Strategic Provisions of Lithuanian Bioeconomy ^4^. However, there are regulations, developmental targets and priorities that directly and implicitly touch upon the topics of bioeconomy and are encompassed in several national strategies and policies that serve as general support for bioeconomy development in Lithuania. Lithuania’s path towards the bioeconomy strategy can be reflected in Figure 1.

Lithuanian National CAP Strategic Plan ^5^ identifies the necessity to pay special attention to the production of goods with high added value using biomass. Strategic priorities included in the Comprehensive Plan of the Territory of the Republic of Lithuania ^6^ cover sustainable economy, resource efficiency, sustainable use of resources, renewable energy sources, climate change mitigation, efficient use of the recreational potential of natural areas and other topics. In the National Energy and Climate Action Plan of the Republic of Lithuania for 2021-2030 ^7^, it is planned that by 2030, biofuel will account for 9% and biogas for 2% of electricity production; also, reorientation of biogas power plants to biomethane gas production and supply of this gas to networks is provided. In the heating sector, the main part will be the heat energy produced from local biofuels. It is planned to reduce greenhouse gases (GHG) emissions in the agricultural sector by 9% in the 2021-2030 period. In the land use, land-use change and forestry (LULUCF) sector, a lot of attention will be paid to the absorption of GHG in the perennial crop biomass by afforestation and promotion of perennial crop production on agricultural land. In the Lithuanian Concept of Scientific Research and Experimental Development and Innovation (Smart Specialization) ^8^, the first and second R&D priorities include several topics directly related to bioeconomy, such as safe food and sustainable agrobiological resources, molecular technologies for medicine and biopharmacy, advanced materials and structures, renewable energy resources, and flexible technology for product development, production and process management and design. There are other documents related to bioeconomy in Lithuania, such as the National Energy Independence Strategy ^9^, the White Paper on Lithuanian Regional Policy ^10^, and the Programme of the Eighteenth Government of the Republic of Lithuania ^11^.

The Ministries of Agriculture and Environment mainly support the primary sectors of the bioeconomy (i.e., agriculture and fisheries, and forestry respectively). Moreover, the Ministry of Agriculture also supports the food manufacturing sector and the Ministry of Environment supports the biowaste management sector. The Ministry of Energy mainly supports the energy sector, including bioenergy. Certain functions of policy-making in bioenergy are also carried out by the Ministry of Agriculture. The Ministry of the Economy and Innovation mainly supports (as a part of policy-making) other bioeconomy sectors. Ministries such as the Ministry of Education, Science and Sport and the Ministry of the Economy and Innovation mainly support (as a policy-making) the bioeconomy-related research and development and innovation (RDI). The Ministry of Agriculture is the coordinating institution of bioeconomy policy in Lithuania.

In Lithuania, many research institutes and universities actively support bioeconomy development by conducting bioeconomy-related research projects. Such research institutes are: Lithuanian Research Centre for Agriculture and Forestry, Institute of Economics and Rural Development of the Lithuanian Centre for Social Sciences, Lithuanian Energy Institute, Nature Research Centre, Centre for Innovative Medicine and universities: Vytautas Magnus University (Agriculture Academy, Faculty of Natural Sciences), Vilnius University (Life Sciences Centre), Kaunas University of Technology (Biomedical Engineering Institute Food Institute, Institute of Mechatronics), Gediminas Technical University (Research Institute of Building Materials), Klaipeda University (Marine Research Institute), and Lithuanian University of Health Sciences (Veterinary Academy). Also, there are private RDI institutions specialised in the bioeconomy in Lithuania, namely: “Thermo Fisher Scientific Baltics”, “Teva Baltics”, and “Biotechpharma”. Enabling RDI Institutions specialized in bioeconomy in Lithuania are: Nemunas Valley, Santara Valley, Santaka Valley, Marine Valley, and “Open R&D Lithuania” network.

There are a few enabling institutions specialised in financial services for bioeconomy or bioeconomy-related sectors in Lithuania: JSC “Agricultural Credit Guarantee Fund”, The Agency for Science, Innovation and Technology, Innovation Agency Lithuania, Research Council of Lithuania, INVEGA, Environmental Projects Management Agency, “Livonia Partners” and “BaltCap” (private equity funds), “LitCapital”, “Business Angels Fund I”, and “Practica Capital” (risk equity funds). Agriculture is the most supported sector of bioeconomy in Lithuania. It is highly dependent on the founding of the common agriculture policy, i.e., on the funding of the European agricultural guarantee fund and the European fund for rural development.

In Lithuania, a lot of attention is paid to bioeconomy-related cluster development. Clusters first appeared in Lithuania several decades ago, but the pace of clusterisation increased dramatically during the period of 2010– 2015, with the implementation of EU financial instruments supporting their development. Clusters specialised in the bioeconomy in Lithuania are: Smart Food Cluster, National Food Cluster, Alliance of Baltic Beverage Industry, Lithuanian prefabricated wooden house cluster – “PrefabLT”, Biopower Plant Development Cluster, Life Sciences Digital Innovation Hub Cluster, “Food Technologies Digitalization LT”, “InnoTekstil”, “Baltic Furniture Cluster”, “ECO Homestead Cluster”, and “Cleantech Cluster Lithuania”.

## Lithuanian bioeconomy profile

### Biomass resources

Lithuania has a high potential of biomass resources from agricultural production and forestry. The latest studies ^4, 12^ show that Lithuania is one of the EU countries that is most self-sufficient in biomass, nevertheless, Lithuania's dependence on biomass imports has been increasing for some time.

**Figure 2.**
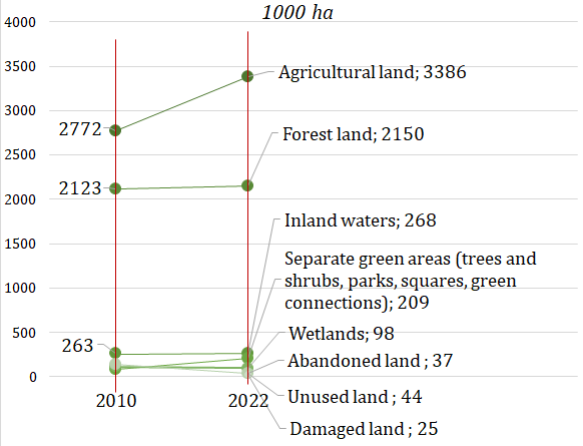
Land areas for biomass production or extraction in Lithuania. Source: Authors’ own composition based on the statistics data of the National land service under the Ministry of Environment of the Republic of Lithuania ^13, 14^.

The main source of production or extraction of biological resources (biomass), but at the same time the limiting factors are soil and water, i.e., land and water body areas. In Lithuania, 3,386 thousand hectares were used for agricultural production in 2022, which accounted for 51.9% of the entire territory of the country, forest land amounted to 2,150 thousand hectares (32.9%), there were also 37.4 thousand hectares of abandoned land (0.6%) and 268.2 thousand hectares inland waters (4.1%). Other potential sources of extraction of biological resources are the area of wetlands – 97.8 thousand hectares (1.5%), separate green areas (parks, squares, green connections) and green areas (trees and shrubs) not classified as forests – 208.6 thousand hectares (3.2%), unused land – 40.7 thousand hectares (0.7%), as well as damaged land – 24.8 thousand hectares (or 0.4%). In 2022, land (agricultural land, forest land, inland waters, abandoned land) suitable for growing biomass accounted for 91% of the country's territory ( Figure 2).

Lately, the country has seen that increasing areas are used in agriculture, forest areas and other land areas (wetlands, separate green areas, unused and abandoned land, as well as damaged land), suitable for growing plant biomass, and this shows the growing potential for the extraction or production of biological resources in Lithuania. On the other hand, the possibilities and limits of sustainable use of Lithuanian land resources for biomass production have not been explored. In Lithuania, inland waters are used very little for aquaculture cultivation.

Currently, biomass accounts for more than a third (on average 35.9%) of all materials consumed in the country and more than a third of all material exports from Lithuania. The direct consumption of biomass in the Lithuanian economy in 2000-2018 increased by almost 69%, i.e., from 17.3 to 29.1 megatons. In the decade after the 2009 global economic crisis, direct consumption of biomass in Lithuania grew much faster (on average by 4.1% per year) than before (on average by 2.9% per year). By contrast, fossil fuel consumption grew at a slower pace after the economic crisis (on average by 1.2% per year) compared to very strong growth in the decade before the crisis (on average by 7.9% per year). This shows that the Lithuanian economy is increasingly reorienting towards the use of biological materials instead of fossils. The main source of biomass in Lithuania is agriculture, which accounts for about 76-80 percent of the total domestic biomass production, followed by wood biomass, which accounts for about one-fifth of the total biomass production in the country, and the share of fisheries and aquaculture is lower than 1% ^1^. In the opinion of Lithuanian experts ^15^, over the past 20 years, the extraction of wood biomass has grown along with the growth of forest stocks.

In Lithuania, more than half (54%) of plant, animal (excluding manure and slurry) and food waste is generated in agriculture and forestry. A lot of food waste is generated in schools, kindergartens, hospitals, prisons and other public catering establishments. The third source of plant and animal waste is the food industry, where animal and plant waste are generated due to technological processes of food and feed production and other losses in manufacturing. Lithuanian agriculture and forestry generate over 10 million tonnes (2018) of plant and animal waste and about three-quarters of it is consumed in the farms and enterprises themselves. Straw and manure account for more than three-quarters of the accounted waste. The farms and companies themselves use most of the straw, horticultural waste, manure, slurry and wood waste, about half of the grain cleaning waste (composting, land treatment or the other way) and they use a small part of other waste. Only a small part (up to 10–15%) of the felling waste remaining at felling sites is collected and used for biofuels and biogas production. Every year, at least 0.4–0.5 million cubic meters of illiquid parts of trees that are left in the forest for natural decay can be used for fuel in Lithuania. Along with this, the restrictions on the removal of felling waste are emphasized. Residues from agriculture and forestry should be used not only for the production of bioenergy by incineration, but also through a variety of tiered solutions that help to create maximum added value. Implementing cascading use, the interaction between the cascading use of biomass and case-by-case externalities, such as emissions, organic carbon loss in the soil or other environmental damage, biodiversity loss or other impacts, should be optimized ^4^.

### Trends in Lithuanian bioeconomy

**Figure 3.**
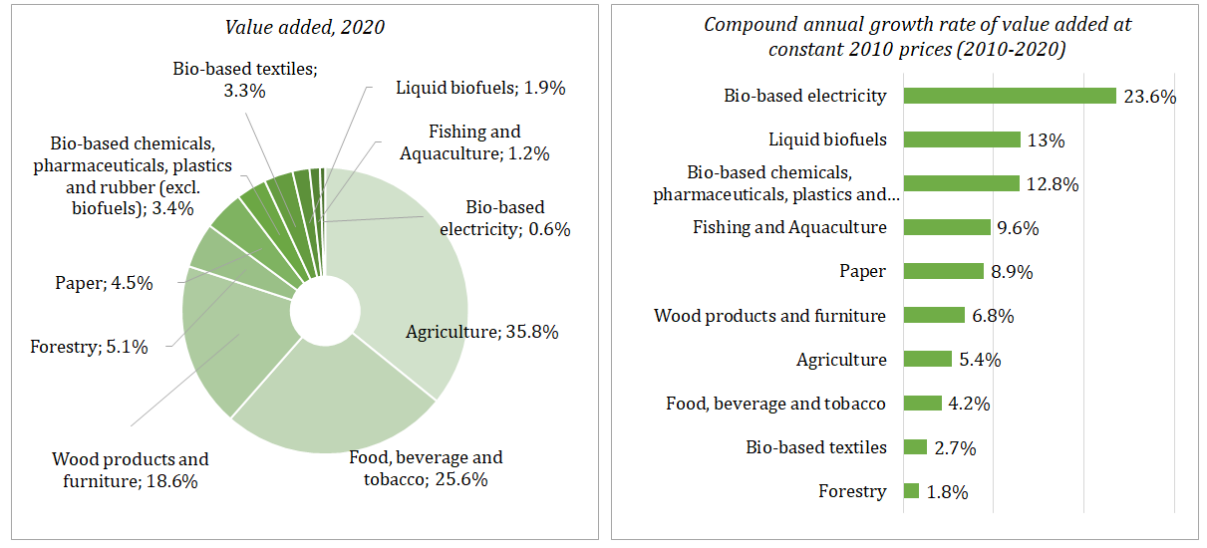
The growth and structure of value added across main industries of Lithuanian bioeconomy. Source: Authors’ own calculation based on data of Data-Modelling platform of resource economics of the European Commission, available at https://datam.jrc.ec.europa.eu/datam/mashup/BIOECONOMICS/index.html. GDP implicit deflator (price index 2010=100) from the Eurostat database [Online data code: NAMA_10_GDP__custom_6394321] was used to measure the real growth of value added.

**Figure 4.**
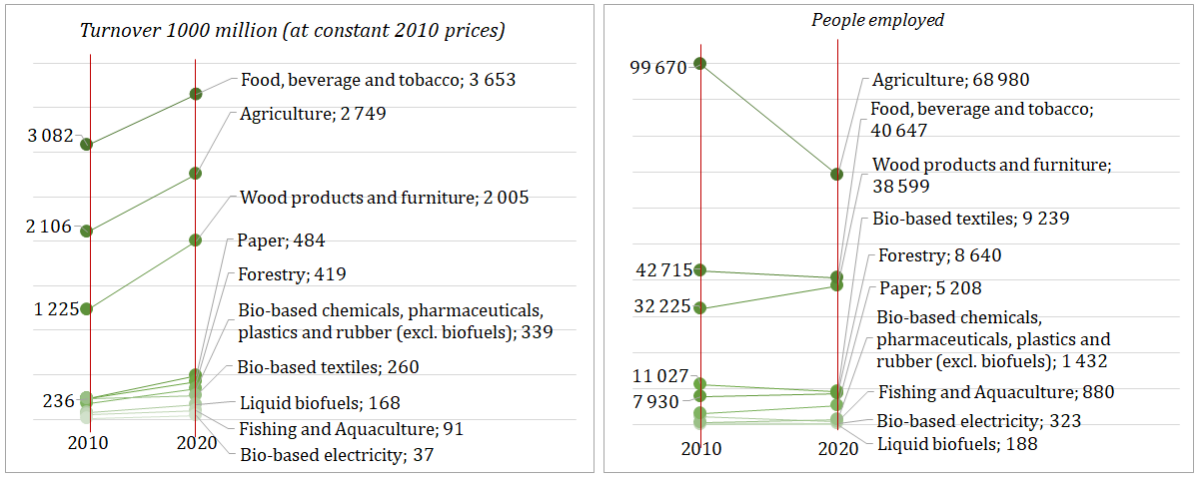
Turnover and employment across main industries of Lithuanian bioeconomy, 2010–2020. Source: Author’s own calculation based on data of the Data-Modelling platform of resource economics of the European Commission, available at https://datam.jrc.ec.europa.eu/datam/mashup/BIOECONOMICS/index.html. GDP implicit deflator (price index 2010=100) from the Eurostat database [Online data code: NAMA_10_GDP__custom_6394321] was used to measure the real growth of turnover.

Lithuanian bioeconomy generated about EUR 4.2 billion of value-added, EUR 12.9 billion turnover, and employed around 174 thousand people in 2020. In the last decade, the share of the bioeconomy in Lithuania’s GDP increased from 6.8 to 8.4%, meanwhile decreased in the labour market (from 16 to 14%) and in the overall turnover of non-financial corporations including agriculture (from 14 to 13%) as well. The agriculture, manufacture of food, beverages and tobacco and the manufacture of wood products and bio-based furniture are the largest bioeconomy industries, altogether generating about 80% of the total value added and 82% of the total turnover in Lithuanian bioeconomy. These three industries provide 85% of the total employment in the Lithuania’s bioeconomy ( Figure 3 and Figure 4).

From 2010 to 2020, the real growth of the bioeconomy was faster than Lithuania’s whole economy by a compound annual growth rate of +5.5% and +3.4%, respectively. Among the bioeconomy industries, the most rapid growth was found in the bio-based electricity – compound annual growth of +23.6% in the same period ( Figure 3). From 2010 to 2020, the highest turnover was observed in the manufacture of food, beverage and tobacco and in agriculture ( Figure 4). The most rapid growth of turnover from 2010 to 2019 was observed in the manufacture of wood products and furniture.

### National energy dependency

**Figure 5.**
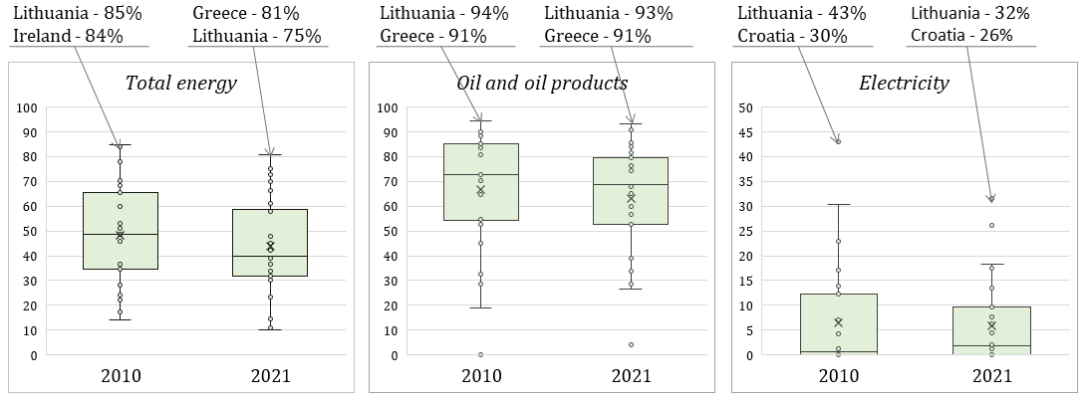
Imports of energy consumed in the EU countries. Source: Authors’ own composition based on Eurostat data on the Energy imports dependency (Online data code: NRG_IND_ID).

The aspiration for energy independence is the driving force of bioenergy development in Lithuania. Figure 5 reflects imports of energy consumed in the EU countries in 2010 and 2021 (only Lithuania and other EU countries with extreme value are distinguished). As shown in Figure 5, Lithuania is a leader in imports of energy consumed (the exception is 2021 when Greece had a higher percentage of imports of consumed energy).

The main insights justify the selection of energy-related bioeconomy sectors for further development:

- Lithuania's high level of energy dependence on imports and challenges to energy security.
- Expensive fossil fuels, as well as emission allowances for CO _2_ emissions and the catching-up energy crisis increase the economic attractiveness of biogas production and the development of this sector.
- The EU's green rate policy and the strategic decisions resulting from it regarding the reduction of the EU's dependence on fossil fuel imports; renewable energy sources (RES), including the development of biogas and advanced biofuels.

Built upon driving forces and barriers to such development towards the bioeconomy, the SWOT framework provides insights on the effective conditions to be met for the transition to bioeconomy. Internal business factors reveal the strengths and weaknesses of a niche sector, while external factors reveal opportunities and threats arising from the related environment. Cross-checking and exploitation of such information after consideration of the current policy coherence overview can lead to explicit strategic actions. This idea is presented in the next sections to derive strategy and action for the Lithuanian biogas and biofuels sectors ^16^.

### The current state of the biogas sector and appropriate strategic actions

In the case of the Lithuanian biogas sector, for many years Lithuania paid the highest price in Europe for gas supplied by Gazprom’s monopoly. Lithuania serves as an example of achieving energy independence from an import monopoly of expensive natural gas over a short period of time ^17^. The production of all types of biogas in Lithuania has grown fast - on average by 25.8% per year in 2009–2018 (from agricultural waste - 36.9% per year, from landfills - 25.8% per year, from wastewater treatment - 13.5% per year). The main raw materials for biogas production are animal manure, rotting organic and food waste from agriculture and processing, etc., which are generated in large quantities in Lithuania ^4^.

The Klaipėda LNG (liquefied natural gas) terminal is one of the most important national energy security facilities, which created the conditions for the emergence of the natural gas market in Lithuania and opened up the opportunity for the state and its citizens to import natural gas from all over the world. Natural gas can be supplied to consumers from different suppliers at market prices. The LNG terminal started operating in December 2014. The LNG terminal consists of a floating storage regasification unit named Independence, a berth and a gas pipeline ^18^.

In 2021, 62% of the total amount of gas supplied to the markets for consumers in Lithuania, the Baltic States and Finland, was imported through the Klaipėda LNG terminal. Most natural gas was imported from the USA through the infrastructure in Klaipėda ^19^. From 1 April 2022, no natural gas from Russia was imported to Lithuania for the needs of the country and other EU countries through the connection of the transmission system with Belarus. From April 2022, the entire amount of gas required for Lithuania's needs is supplied through the transmission system from the Klaipėda LNG terminal and Latvia ^20^.

The development of biogas production from agricultural waste in Lithuania has gained significant acceleration only since 2014, when UAB Modus energy-built biogas power plants with a capacity of 0.5–1 MW in 8 pig complexes. Currently, 14 power plants extract biogas from agricultural waste, 10 of which process animal manure. These power plants use only about 2% of animal manure or about 20% pig manure. A large part of manure – even 85% – is produced on livestock holdings, but no power plants have been built on these holdings. The production of alternative energy from manure and other waste should be prioritized over other forms of alternative energy production, but the production of biogas from manure is lagging behind in terms of the amount of energy produced. About 11 million tons of manure are generated in Lithuania every year. According to the data of 2015, the use of manure for biogas production (about 30%, 3.3 million tons) would reduce GHG emissions from manure by 140 times, calculated in carbon dioxide (CO _2_) equivalent, and without changing emissions from other sources, emissions in agriculture would decrease by about 3% ^21^.

The most efficient way to increase biogas production is to recycle organic waste such as cattle, pig and poultry manure. However, despite rapidly improving technologies, the potential of raw materials for biofuel production in Lithuania is not properly exploited. The largest untapped potential of raw materials lies in straw, manure, meadows and municipal waste. Research shows that pure diesel from renewable sources emits 60–85% less greenhouse gases than conventional diesel. Particulate emissions are reduced by 30-40% and nitrogen oxides by 10%. In addition, biomethane, as a second-generation biofuels, is the most cost-effective way to achieve the set goals of share of renewable energy in the transport sector ^22^.

In Lithuania, the share of biogas transformed into electricity is growing every year. In 2018 more than two-thirds (78%) of produced biogas was used in thermoelectric power plants ^4^. The national energy independence strategy provides for an increase in the share of electricity consumption from RES up to 45% in 2030 and 80% in 2050 compared to the final electricity consumption. Taking into consideration the assessment of the technology development trends, by 2025, it is estimated that 15% of RES-generated electricity could come from biofuel and about 3% from biogas, while by 2030, biofuels could generate about 16% of electrical energy and biogas about 1% ^9^.

It is planned to promote gaseous biofuels (biomethane) use in public transport. It is foreseen in the Alternative fuels law (2021) to reduce the transport sector’s impact on climate change and air pollution through increasing RES use in the sector by no less than 15% by 2030 and 3.5% should be coming from advanced biofuels and/or non-biological liquid and gaseous fuels from RES ^23^. As stipulated in the Alternative fuels law, biogas or non-biological gaseous fuels from RES must account for at least 16.8% of the energy value of gas supplied to the transport sector in the internal market by 2030. While it is forecasted to experience supply shortages of advanced biofuels, experts see a great potential of biomethane, produced from agriculture and manufacturing waste, in Lithuania.

The development of the biogas production sector is of interest not only to the businesses operating in this sector, but also to society, represented by the state government. The biogas production sector contributes to the accommodation of public interests, i.e., reduction of dependence on fossil fuels, mitigation of climate change and reduction of its negative effects, sound and sustainable economic development.

The main public goals of Lithuanian society in relation to the development of the biogas production sector until 2030 are declared in the National Climate Change Management Agenda of Lithuania (2021) ^24^ approved by the Parliament of the Republic of Lithuania, harmonized with the main strategic documents of the EU Green deal. Biogas production is carried out according to the principles of the circular economy and reduces greenhouse gas emissions. Therefore, the biogas production sector also contributes to the achievement of general climate change goals. Until 2030 in agriculture alone, it is aimed to reduce greenhouse gas emissions by at least 11% compared to 2005 and achieve that 50% pig and cattle manure would be used to produce biogas ^24^.

The main internal strength factors of the Lithuanian biogas sector are:

- High potential of sewage, agricultural, municipal, and industrial waste that is suitable for biogas production.
- Acceleration of biogas production adoption in wastewater treatment plants and large pig farms.
- Implementation of administrative instruments for the recognition of guarantees of origin for green gas (National Register of Guarantees of Origin for Gas Produced from RES; Accounting System for Fuels from Renewable Energy Sources).
- Biogas production from plant waste, manure and slurry follows the principles of a circular economy and reduces GHG emissions.
- The country's biogas production equipment industry is growing, providing biogas power plants and biogas purification (biomethane production) equipment to the market.
- A Biopower Plants Development Cluster operates in the country, uniting businesses, agricultural sector companies, research, and study institutions for the development of biomethane production technologies and the dissemination of information in society.

The main harmful internal factors (weaknesses) of the Lithuanian biogas sector are:

- In terms of biogas production capacity, Lithuania is a lagging country in the EU.
- No production of biogas on livestock farms, underdeveloped production of biogas from poultry manure.
- Due to large investments, farmers and agricultural enterprises have no interest in adopting and developing biogas production.
- Infrastructure for the supply of biogas to natural gas networks is underdeveloped. Produced biogas is used exclusively for producers' internal needs (electricity and heat production).
- The collection and use of food waste from the population for biogas production is underdeveloped.
- The conditions for receiving state support do not always meet the technical possibilities of efficient biogas production and the expectations of potential biogas producers.
- The scepticism of local communities towards the construction of new biogas plants is still evident.

The main opportunities for the Lithuanian biogas sector are:

- The rise in the price of natural gas and other fossil fuels, as well as the increase in CO _2_ emissions, and the increase of the economic attractiveness of biogas production and the development of the sector.
- Large amounts of agricultural waste for the rapid development of biogas production. Possibility to produce biogas from biomass waste not used for food and feed (meadow grass, cereal straw, etc.).
- EU (and Lithuanian) Green Deal policy and its strategic decisions on reduction of dependence on fossil fuels, and the development of RES, including biogas.
- Grants and other financial support for the development of biogas production from EU and national funding sources.
- Wider opportunities for the use of biogas (for the decarbonisation of the transport sector; trade in international markets, etc.).
- EU (and Lithuanian) policy on food waste reduction and strategic decisions on its use in biogas production.

The main harmful external factors (threats) in the biogas sector of Lithuania are:

- The declining scale of agriculture in the country, especially livestock farming, may adversely affect the potential of a raw material suitable for biogas production.
- Limited opportunities to develop biomethane supply systems to natural gas supply networks (infrastructure). Limited number of suitable sites to connect biomethane production facilities.
- Potentially increasing competition with other RES production methods, as well as biogas imports from other European countries.
- Lack of opportunities for cooperation and community interaction in organizing the supply of biogas power plants with raw materials, developing local biogas use systems, and collecting waste from the small farms prevailing in the country.
- Threats to the establishment of stable and effective state support for the development of biogas production and consumption.

SWOT elements can then be elaborated and classified into internal and external factors plotted against the opportunities and threats axis. The TOWS matrix derived elaborates upon: use strengths to maximize the identified opportunities and/or minimize the effect of threats, to minimize the weaknesses using the identified opportunities and/or to avoid the identified threats. Taking into account the results of the analysis in the Lithuanian biogas production sector, the following strategic directions for the intervention in this sector have been identified:

- *First direction.* Provide state financial support for the investments of farmers, agricultural companies, and other companies in biogas power plants, biomethane purification and conversion into heat and electricity capacities. When providing investment support, it is appropriate to give priority to commercial biomethane production, as well as to biogas power plants cooperatively supplied with raw materials from surrounding small farms or integrated into local biogas utilization systems.
- *Second direction*. Develop biomethane supply infrastructure by adapting natural gas networks for biomethane transmission, enabling biomethane producers to connect to the networks. Potential connections to natural gas networks and expanded biomethane sales opportunities must be inventoried and expanded.
- *Third direction.* Provide state financial support to municipalities and communities to establish systems for food waste collection and use for biogas production. When providing support, it is appropriate to give priority to integrated biogas production systems that include the use of not only food but also biomass waste of other origins.
- *Fourth direction.* Provide support for information dissemination, training, and consulting projects aimed at improving community awareness of the biogas benefits, its production technologies and local systems, environmental impact, and other important aspects of biogas production and consumption.

### The current state of the biofuels sector and appropriate strategic actions

In the case of the biofuels sector in Lithuania, firewood and wood waste are traditionally used as the main carriers of bioenergy, and the development of the use of other types of biomass in energy began in 2002, when agricultural waste was started to be used for energy production and biogas was started to be produced from sewage sludge. In 2003, biogas started to be produced from agricultural waste, and since 2004 bioethanol and biodiesel have been produced. Lithuania produces two types of biofuels: biodiesel and bioethanol. The main feedstocks for biodiesel and bioethanol production are rapeseed and rye. About 500,000 tons of grain and rapeseed are purchased for biofuels production every year. During the last five years (2016–2021), the processing of rapeseed into biodiesel grew by 7.5%, the processing of grain into bioethanol grew by 4.3% per year ^2^. During the same period, the area of rapeseed crops doubled, and the yield grew by an average of 23% per year, while, cereal yields remained stable, despite a slight decrease in the area under cereal crops ^3^.

The association of biofuels producers consists of 6 members, the main producer of biodiesel is "Rapsoila" and produces bioethanol, "Kurana". The latter company, in 2019 started to produce the advanced biofuels and plans to produce 1.2 thousand cubic meters of advanced bioethanol from by-products, and eventually increase its production capacity to 5,000 cubic meters ^25^.

In Lithuania, there is a large amount of waste suitable for the production of biofuels. The absorption of the production of advanced biofuels requires large investments and logistical systems for the collection of raw materials. The Government of Lithuania has included a measure in the DNA plan for the future economy of Lithuania, which is intended to finance the increase of production capacity of advanced biofuels. One million euros would be allocated from DNA funds for this measure. The support concept envisages the promotion of advanced bioethanol and biodiesel production capacities by utilizing the potential of raw materials created in Lithuania. Production of advanced biofuels is extremely necessary to achieve ambitious GHG reduction goals and decarbonization of the transport sector ^26^. 

Based on our analysis of Lithuania’s bioenergy data ^4^, the total consumption of biofuels in Lithuania has been growing rapidly over the past decade - 2.5 times increase in 2010–2021. In the same period, the export of biofuels also grew and doubled in size. However, the imports of biofuels increased more than 8 times. Nevertheless, palm oil, other low-quality imported biofuels and some synthetic fuel additives are still used to produce diesel, which are significantly cheaper than Lithuanian biodiesel and bioethanol. Therefore, manufacturers of automotive fuels often choose foreign substitutes and increase the import of biofuels.

Lithuania's National Energy Independence Strategy (2018) envisages a goal of 15% of RES share in the final consumption of the transport sector for 2030, the majority of which is planned to be achieved precisely through the promotion of the use of biofuels. Using advanced biofuels in vehicles emits on average up to 90% less GHG compared to using the same amount of mineral oil fuel ^9^.

Not only entrepreneurs operating in this sector, but also the society, represented by the state government, are interested in the development of the biofuels production sector. The biofuels production sector contributes to the satisfaction of public interests, that is, the reduction of dependence on fossil fuels, mitigation of climate change and reduction of its negative effects, and sustainable and harmonious economic development. The main public interests of the Lithuanian society in relation to the development of the biofuels production sector until 2030 are declared in the National Climate Change Management Agenda of Lithuania ^24^, reconciled with the main strategic documents of the EU Green Deal. This agenda envisages promoting the production of advanced biofuels and achieving that they constitute at least 3.5% of the final energy consumption of the transport sector until 2030.

In summary, it can be said that the public interest of Lithuania in the biofuels production sector is to develop the production and consumption of advanced biofuels on the principle of circularity, reducing dependence on fossil fuels and contributing to climate change management.

The main internal strength factors in the Lithuanian biofuels sector are:

- The great potential of the country's biological resources and its improving use for biomass extraction, especially in agriculture.
- Long-standing traditions of grain and rapeseed cultivation, application of innovative technologies and increasing productivity.
- Developed industry and acquired experience in the production of biofuels from agricultural raw materials (starchy and oilseed products that can be used for food and feed).
- Implemented legal framework for biofuels production and consumption in line with EU legislation and methodology for biodiesel quality assessment.
- The biofuels industry has the potential to meet the country's domestic needs for biofuels as an additive to fossil fuels and to increase their exports to European countries.
- Technologies used in the production of biofuels from agricultural raw materials are based on the principles of a circular economy and are environmentally friendly.
- High potential for waste and residues suitable for the production of advanced biofuels.

The main harmful internal factors (weaknesses) of the Lithuanian biofuels sector are:

- Attachment to the production of biofuels from agricultural raw materials. Relatively low investment in the production of advanced biofuels.
- Biofuels production is export-oriented and poorly adapted to produce more advanced biofuels for the country’s domestic needs.
- The rapid increase in cereal production volumes, including for biofuels production, is inharmonious.
- According to the market price, the competitiveness of Lithuanian biofuels in terms of fossil fuels and palm oil is low.

The main opportunities for the Lithuanian biofuels sector are:

- EU and national legislation promotes the transition from agricultural feedstocks to the use of biowaste and residues in biofuels production by supporting advanced biofuels producers and research.
- Increasing use of animal fats, used oils and cooking oils and other food waste, as well as algae in the production of advanced biofuels.
- Rapid development of research into advanced biofuels feedstocks, production and use technologies.
- International agreements and EU (and Lithuanian) strategic decisions on mitigation of climate change may lead to restrictions on the use of palm oil and other environmentally unfriendly biofuels.
- Opportunities for local biofuels production to meet the decarbonisation targets for the transport sector set out in EU and national legislation.
- Possibility to produce biofuels for aviation (Sustainable Aviation Fuels).

The main harmful external factors (threats) in the Lithuanian biofuels sector:

- EU and national legislation promotes the transition from agricultural feedstocks to the use of biowaste and residues in biofuels production by supporting advanced biofuels producers and research.
- Increasing use of animal fats, used oils and cooking oils and other food waste, as well as algae in the production of advanced biofuels.
- Rapid development of research into advanced biofuels feedstocks, production and use technologies.
- International agreements and EU (and Lithuanian) strategic decisions on mitigation of climate change may lead to restrictions on the use of palm oil and other environmentally unfriendly biofuels.
- Opportunities for local biofuels production to meet the decarbonisation targets for the transport sector set out in EU and national legislation.
- Possibility to produce biofuels for aviation (Sustainable Aviation Fuels).

Taking into account the results of the analysis of the Lithuanian biofuels production sector based on the SWOT and TOWS methodology, the following strategic directions of intervention in this sector have been identified:

- *First direction.* Rapidly develop the production of advanced biofuels from biological wastes and residues, including animal fats, used oils and cooking oils and other food waste, as well as algae, and use in the transport sector. It is appropriate to provide financial support: i) for scientific research projects in the fields of advanced biofuels raw materials, production and use technologies; ii) to create and implement collection and logistics systems for biological waste and residues, including fats of animal origin, used oils and cooking oils and other food waste, as well as algae; iii) for investments in advanced biofuels production capacities.
- *Second direction.* To improve the legal regulation of biofuels production and consumption as fuel. Legislation is appropriate to provide for the restriction of the use of palm oil and other environmentally unfriendly fuels.

## Conclusions

A synthesis of the aforementioned sectoral strategic directions is necessary to develop over-arching national bioeconomy-appropriate strategic actions that can be summarized in three areas with regard to:

Market intervention actions:

- Promote the creation of a competitive environment for business development, also move from commodity to products with higher added value through implementation of innovative technologies and business models for prevention, reuse, recycling and use of biological waste.
- Provide financial support to businesses, municipalities and clusters for implementing scaling up of innovative technologies.
- Public investment in infrastructure (biomethane distribution network, district heating, etc.).

Research, innovation and education:

- National research agenda is a functional part of the BIOEAST macro-region’s Strategic Research and Innovation Agenda (SRIA).
- The need for actions to improve Lithuania’s bioeconomy-related research and innovation can be resumed in:
  • *Support (financial and nonfinancial):* plan the consistent funding for the research, based on medium and long-term strategic planning; provide better support for business innovation by upgrading competences of science, technology and innovation public sector policymakers; etc.
  • *Cooperation:* enhance integration with international innovation networks; promote the development of bioclusters; etc.
  • *Innovations:* implement the circular principles throughout the food supply chain; ensure sustainable agriculture activity in a circular way by using locally available and produced resources; etc.
  • *Knowledge transfer, education:* promote the development of bioeconomic hubs and networks to ensure learning from best practices; enhance understanding of bioeconomy at the business, scientific, governmental and consumer levels, better use the education system at all levels; etc.

Governance and policy actions:

- Set up a Bioeconomy Council to ensure long-term engagement at the national level to act as a catalyser for interministerial and interinstitutional coordination;
- Enforce the relationship between the main sub-sectors of bioeconomy and niche sectors in the framework of bioeconomy strategy or action plan;
- Elaborate legal regulations to enable a predictive environment in the bioeconomy.

## Ethics and consent

Ethical approval and consent were not required.

## Data Availability

No data are associated with this article.

## Footnotes

^1^ Authors’ own calculation based on the Eurostat data of Material flow accounts [online data code: ENV_AC_MFA]

^2^ The authors’ calculation is based on statistical information provided on our individual request by the Lithuanian Department of Statistics.

^3^ The authors’ calculation is based on data from the Lithuanian official statistics portal database [online data: Harvested area of agricultural crops and Harvest of agricultural crops from the Crop production database]

^4^ The authors’ calculation is based on data from the Lithuanian official statistics portal database [online data: Fuel commodities balances from the Annual energy indicators database]
