# Supplementary material for: The strategic concept for the Lithuanian bioeconomy: insights for niche bioenergy sectors
Source: Open Res Eur. 2023 Aug 15;3:101. Originally published 2023 Jun 20. [Version 2] doi: 10.12688/openreseurope.16085.2 (PMC10445903; doi:10.12688/openreseurope.16085.2)
Supplement: Peer review history

# Reviewer report: Reviewer response for version 2

**DOI:** 10.21956/openreseurope.17735.r34428

**Published:** 2023-10-18

**Authors:** Rolandas Urbonas

**Affiliations:** 1 Lithuanian Energy Institute, Kaunas, Lithuania

Dear Authors,

 Thank you very much for your updated manuscript.

 My only comment and suggestion would be to elaborate more on Figure 5 so that non-experts readers were more comfortable. 

 The dependency from third countries is not necessarily a bad situation, since among the third countries we see (in terms of energy exporters) Norway, USA.

 I wish the Authors every success in their research.

Where applicable, are recommendations and next steps explained clearly for others to follow? (Please consider whether others in the research community would be able to implement guidelines or recommendations and/or constructively engage in the debate)

Yes

Does the article adequately reference differing views and opinions?

Yes

Are all factual statements correct, and are statements and arguments made adequately supported by citations?

Partly

Is the rationale for the Open Letter provided in sufficient detail? (Please consider whether existing challenges in the field are outlined clearly and whether the purpose of the letter is explained)

Yes

Is the Open Letter written in accessible language? (Please consider whether all subject-specific terms, concepts and abbreviations are explained)

Yes

Reviewer Expertise:

Energy

I confirm that I have read this submission and believe that I have an appropriate level of expertise to confirm that it is of an acceptable scientific standard.

# Reviewer report: Reviewer response for version 1

**DOI:** 10.21956/openreseurope.17367.r32990

**Published:** 2023-07-19

**Authors:** Vida Dabkienė

**Affiliations:** 1 Lithuanian Centre for Social Sciences, Vilnius, Lithuania

The article deals with one of the most relevant topics in Lithuania - the current situation of the bioenergy sector, its development and SWOT analysis. The authors reveal their deep knowledge and expertise by describing in detail the bioeconomy-related institutional framework in Lithuania. The authors separately analyse the situation regarding biogas and biofuels and present strategic actions. The paper tackles one of the drivers of bioeconomy development, i.e. the independence of the energy sector. The authors illustrate the situation well in Figure 5. The paper demonstrates a solid knowledge of statistics, scientific and grey literature on the bioeconomy sector, giving the case of Lithuania. The SWOT analysis is an essential part of the strategic plan and is a powerful tool in the strategic planning process. The strategic orientations for biogas and biofuels presented here are one of the most significant outputs of this work and a contribution to the development of the country's bio-economy sector, with a view to creating a comprehensive national bio-economy.

Where applicable, are recommendations and next steps explained clearly for others to follow? (Please consider whether others in the research community would be able to implement guidelines or recommendations and/or constructively engage in the debate)

Yes

Does the article adequately reference differing views and opinions?

Yes

Are all factual statements correct, and are statements and arguments made adequately supported by citations?

Yes

Is the rationale for the Open Letter provided in sufficient detail? (Please consider whether existing challenges in the field are outlined clearly and whether the purpose of the letter is explained)

Yes

Is the Open Letter written in accessible language? (Please consider whether all subject-specific terms, concepts and abbreviations are explained)

Yes

Reviewer Expertise:

Sustainability and competitiveness of agriculture

I confirm that I have read this submission and believe that I have an appropriate level of expertise to confirm that it is of an acceptable scientific standard.

# Response

**Published:** 2023-07-19

**Authors:** Akvile Aleksandraviciene

We thank the reviewer for this detailed and positive assessment of our manuscript and our knowledge.

# Reviewer report: Reviewer response for version 1

**DOI:** 10.21956/openreseurope.17367.r32992

**Published:** 2023-07-18

**Authors:** Magdalena Borzęcka

**Affiliations:** 1 Institute of Soil Science and Plant Cultivation – State Research Institute, Puławy, Poland

The article broadly describes the strategic concept for the development of bioeconomy in Lithuania. The work was carried out in the international project BIOEASTsUP H2020. The article presents a conceptual basis that can be used to prepare a national bioeconomy strategy and/or action plan to initiate discussion on the strategically oriented development of a knowledge-based circular bioeconomy in Lithuania. The paper concept paper focuses on two niche energy sectors, such as biofuels and biogas. The presented results of the SWOT analysis show that in Lithuania, the synthesis of the strategic directions of the biofuels and biogas sectors is necessary to develop overarching national strategic actions relevant to the bioeconomy development, such as market interventions, research, innovation and educational activities, as well as governance and policy actions. The paper describes the related institutional framework in Lithuania, indicating plans, public institutions, scientific units and institutions specialized in financial services as well as clusters that may have an impact on the development of the strategy. The next chapter describes biomass resources in Lithuania, possibilities and changes in its use to reduce high energy dependency. The suggested solution is to strengthen the position of biogas and biofuels. The work discusses in detail SWOT elements, i.e. strength factors, main harmful internal factors, main opportunities, and threats. The summary shows the possibility of introducing interventions on the market as well as the paths for research, innovation and education.

 The work was done very carefully, with well-chosen literature.

Where applicable, are recommendations and next steps explained clearly for others to follow? (Please consider whether others in the research community would be able to implement guidelines or recommendations and/or constructively engage in the debate)

Yes

Does the article adequately reference differing views and opinions?

Yes

Are all factual statements correct, and are statements and arguments made adequately supported by citations?

Yes

Is the rationale for the Open Letter provided in sufficient detail? (Please consider whether existing challenges in the field are outlined clearly and whether the purpose of the letter is explained)

Yes

Is the Open Letter written in accessible language? (Please consider whether all subject-specific terms, concepts and abbreviations are explained)

Yes

Reviewer Expertise:

Bioeconomy, Biomass assesment, Bioefuels

I confirm that I have read this submission and believe that I have an appropriate level of expertise to confirm that it is of an acceptable scientific standard.

# Response

**Published:** 2023-07-19

**Authors:** Akvile Aleksandraviciene

We thank the reviewer for taking the time to provide a review of our paper and for the positive assessment of our manuscript. Your efforts are much appreciated.

# Reviewer report: Reviewer response for version 1

**DOI:** 10.21956/openreseurope.17367.r32989

**Published:** 2023-07-12

**Authors:** Rolandas Urbonas

**Affiliations:** 1 Lithuanian Energy Institute, Kaunas, Lithuania

The paper discusses the current situation in bioeconomy sector in Lithuania - current situation and possible future actions. The institutional framework, bioeconomy profile/statistics and future strategic actions are presented. 

 Even though the thorough overview and analysis are presented, I would have some comments on the text:

1. In the section "Bioeconomy-related institutional framework in Lithuania" the Agency for Science, Innovation and Technology is presented as institution specialised in financial services for bioeconomy or bioeconomy-related sectors in Lithuania. Unfortunately, since the end of 2022 this agency does not exist (functions given to the Research Council of Lithuania and  Innovation Agency).
2. Also I doubt the sentence "Enabling RDI Institutions specialized in bioeconomy in Lithuania are: Nemunas Valley, Santara Valley, Santaka Valley, Marine Valley, and “Open R&D Lithuania” network." Listed institutions may operate as networks of RDI institutions, but not as institutions themselves. 
3. Why are some statistics of the year 2018, while others - the year 2020? Could unified statistics be presented?
4. Could Authors explain the sentence in the section "The current state of the biogas sector and appropriate strategic actions" - "Taking into consideration the assessment of the technology development trends, by 2025, it is estimated that 15% of RES-generated electricity could come from biofuel and about 3% from biogas, while by 2030, biofuels could generate about 16% of electrical energy and biogas about 1% ^9^." Does it mean that in 2030 in comparison to 2025 less electricity will be produced by biogas?
5. It could be the format of the referencing. However the reference in the 2nd paragraph of the section "Introduction" is different than others.
6. I doubt the correctness of Figure 5 (imports of electricity). Since the shutdown of the Ignalina Nuclear Power Plant, Lithuania is an electricity importer country. Electricity import reaches 60-70% ^1^.
7. I am not sure whether it is necessary to mention the Task (number) of the project in the paper.

Where applicable, are recommendations and next steps explained clearly for others to follow? (Please consider whether others in the research community would be able to implement guidelines or recommendations and/or constructively engage in the debate)

Yes

Does the article adequately reference differing views and opinions?

Yes

Are all factual statements correct, and are statements and arguments made adequately supported by citations?

Partly

Is the rationale for the Open Letter provided in sufficient detail? (Please consider whether existing challenges in the field are outlined clearly and whether the purpose of the letter is explained)

Yes

Is the Open Letter written in accessible language? (Please consider whether all subject-specific terms, concepts and abbreviations are explained)

Yes

Reviewer Expertise:

Energy

I confirm that I have read this submission and believe that I have an appropriate level of expertise to confirm that it is of an acceptable scientific standard, however I have significant reservations, as outlined above.

## References

[1] Lithuania Electricity Security Policy. *IEA* .2022; Reference source

# Response

**Published:** 2023-07-19

**Authors:** Akvile Aleksandraviciene

We would like to thank the reviewer for the thorough assessment of our manuscript and for the comments that helped to improve the quality of this article. Regarding your comments and questions, *we have made corrections in the text and updated the analysed data based on the latest available statistical information. We upload version 2 of our manuscript. We have answered the comments in order of appearance below.* 

*1. We have added additional information about the Agency for Science, Innovation and Technology in the text based on your comment. In our opinion, since this Agency ended at the end of 2022, however, it had a great impact on the development of Lithuanian bioeconomy-related RDI within the last few decades. Thus, in our opinion, it is important to mention this Agency.*

 2. Regarding your comment about the valleys as networks, we have made corrections in the text based on the latest available information about the valleys and their activity.

 3. We have taken into account your comment for the unified statistics and we have provided new data for 2020 and 2022 in our analysis according to the latest available statistical information.

 4. The percentage targets of the share of biogas in electricity production are specified in the National Energy Independence Strategy (2018). In our opinion, the reduction of this percentage of the share in 2030 in comparison with 2025 is reasonable since biogas is predicted to be used for other domestic needs than the production of electricity. For instance, as AB Amber Grid argues, biomethane can be used as a substitute for natural gas and fed into gas transportation networks. The REPowerEU plan published by the European Commission aims for the EU to produce 35 billion cubic meters of biomethane per year in 2030, which means that more gas will be used for biomethane extraction and supply to gas transportation networks (see more https://www.ambergrid.lt/zaliosios-dujos/biometanas/33).

 5. We have made corrections in the reference in the 2nd paragraph of the section "Introduction". We have unified the format of the referencing and provided the number of quoted pages in the description of reference No 2.

 6. Regarding your comment about the correctness of Figure 5, we have provided a more detailed explanation of Lithuania’s energy import dependency in the text and the title of Figure 5. This figure reflects Lithuania’s energy dependency on imports from third countries. Based on our calculation, we wrote notes under Figure 5 on Lithuania's overall dependence on electricity imports from all partners, i.e., EU member states and third countries.

 7. The content and the structure of the article were determined by the Task 1.5 in the BIOEAST‘sUP H2020 project. In addition, Open Research Europe is an open access publishing platform for the publication of research stemming from Horizon 2020, Horizon Europe and/or Euratom funding. Therefore, we think, it is important to mention the Task of the project.
